# Adjunctive Therapies During Extracorporeal Membrane Oxygenation to Enhance Multiple Organ Support in Critically Ill Children

**DOI:** 10.3389/fped.2018.00078

**Published:** 2018-04-03

**Authors:** Marguerite Orsi Canter, Jessica Daniels, Brian C. Bridges

**Affiliations:** Division of Pediatric Critical Care, Department of Pediatrics, Vanderbilt University School of Medicine, Nashville, TN, United States

**Keywords:** extracorporeal membrane oxygenation, continuous renal replacement therapy, therapeutic plasma exchange, adsorptive therapies, extracorporeal liver support

## Abstract

Since the advent of extracorporeal membrane oxygenation (ECMO) over 40 years ago, there has been increasing interest in the use of the extracorporeal circuit as a platform for providing multiple organ support. In this review, we will examine the evidence for the use of continuous renal replacement therapy, therapeutic plasma exchange, leukopheresis, adsorptive therapies, and extracorporeal liver support in conjunction with ECMO.

## Introduction

In 1975, Bartlett et al. supported a newborn with refractory hypoxemic respiratory failure and pulmonary hypertension with extracorporeal membrane oxygenation (ECMO) (1). The success of this case led to a revolution in the support of the patient with refractory, but reversible respiratory and/or cardiac failure. Since then, there has been a great expansion in the types of patients supported with ECMO. We are now placing larger numbers of increasingly sick patients on ECMO (2). Once relegated to neonates, adults now comprise the largest group of patients supported with ECMO (3). Previously, there were strict contraindications to ECMO, but now, it is commonplace to place patients on ECMO with septic shock (4), in active cardiac arrest (5, 6), and with irreversible heart or lung failure as a bridge to transplant (7, 8). As we place more complicated patients on ECMO with multiple organ dysfunction, we are increasingly providing multiple organ support. In the neonatal and pediatric population, patient size is a limiting factor in obtaining adequate vascular access. However, in patients supported with ECMO, the extracorporeal circuit provides a platform in which other forms of organ support can be added. In this review, we will look at some of the evidence for providing multiple organ support in conjunction with ECMO.

## Continuous Renal Replacement Therapy (CRRT)

Acute kidney injury (AKI) and fluid overload are commonplace in critically ill patients requiring ECMO. Using the RIFLE criteria, previous studies found an incidence of AKI in ECMO patients of approximately 70% (9–11). A recent, multicenter study using the Kidney Disease Improving Global Outcomes consensus definition found AKI to occur in 74% of children supported on ECMO. For these neonatal and pediatric ECMO patients, AKI was strongly associated with increased duration of ECMO and increased mortality (12). The negative impact of AKI, fluid overload, and need for renal support therapy (RST) on morbidity and mortality has been demonstrated in multiple pediatric and adult studies (13–16).

The pathophysiology of AKI in ECMO patients is complex and multifactorial. Although the literature on the etiology and association of AKI and ECMO is limited, the process is likely driven by pre-ECMO morbidity and exacerbated by intrinsic ECMO factors. Critically ill patients requiring ECMO support are at a high risk for AKI prior to initiation of ECMO due to their underlying pathophysiology (hypoxic insult, low cardiac output state, activation of inflammatory mediators) and the common administration of nephrotoxic medications. Inotropes/vasopressors are often needed to support patients both before and around the time of ECMO initiation, and the use of these agents in critically ill patients is associated with an increased risk of AKI (17). In a multicenter study of AKI in pediatric patients who suffered cardiac arrest, time to return of spontaneous circulation was not associated with the development of AKI, but the total number of epinephrine doses given was associated with the development of AKI (18). The initiation of ECMO can then exacerbate the initial insult by provoking reperfusion injury (19) and exacerbation of fluid overload. Another proposed etiology for AKI in patients on ECMO is the non-pulsatile flow while on venoarterial ECMO (20). However, this is a topic of debate. Adademir et al. found lower IL-18 and neutrophil gelatinase-associated lipocalin (NGAL) levels in adults who underwent pulsatile flow on cardiopulmonary bypass (both markers of renal injury) compared to non-pulsatile flow (21), but there is a paucity of data regarding clinical development of AKI using these different modalities. Additionally, the use of venovenous ECMO, which preserves pulsatile flow, is also frequently complicated by AKI (22). This suggests that the lack or decrease of pulsatile flow is not necessarily a key factor in the development of renal injury. There are some factors intrinsic to ECMO that can also aggravate AKI. The systemic inflammation caused by blood exposure to artificial surfaces exacerbates the likely pre-existing stress response and can cause renal inflammation and injury (20, 23). Another aggravating factor is the common development hemolysis. Elevated levels of plasma-free hemoglobin have been associated with the development of hemoglobinuria nephropathy (20, 24). A large multicenter report on the incidence of AKI in pediatric patients on ECMO showed that the development of AKI occurs early in the ECMO course with 51–64% of patients meeting AKI definitions at initiation of ECMO and 86–93% of AKI developing by 48 h (12), suggesting that significant renal injury has already occurred at the time of ECMO initiation.

Continuous renal replacement therapy is commonly used in critically ill patients as a method of solute clearance and treatment of fluid overload that can be tolerated even in patients with hemodynamic instability. Indications for CRRT in patients on ECMO are similar to classic CRRT indications; electrolyte abnormalities, uremia, and fluid overload. However, a survey of ELSO centers on initiation of CRRT showed significant variation among centers, with 23% of centers reporting no use of RST for ECMO patients (25). This survey also revealed that fluid overload is the most common indication for CRRT (43%). Fluid overload has been shown to be a risk factor for increased mortality and prolonged ECMO duration (26–29). A multicenter study of 756 neonatal and pediatric ECMO patients demonstrated that both degree of fluid overload at ECMO initiation and peak fluid overload during ECMO were independently associated with increased mortality. In survivors, both fluid overload at ECMO initiation and peak fluid overload during ECMO were independently associated with increased duration of ECMO (30).

There are multiple modalities to provide CRRT for patients on ECMO. The three most widely used methods are: introducing an in-line hemofilter into the ECMO circuit, introducing a commercially available CRRT device in the ECMO circuit, and performing CRRT *via* independent venous access (31). In the survey study by Fleming et al., of the responding centers that use CRRT with ECMO, 21.5% of centers exclusively used an in-line hemofilter and 50.8% of centers exclusively used a commercially available CRRT device connected to the ECMO circuit (12).

**Figure 1:**
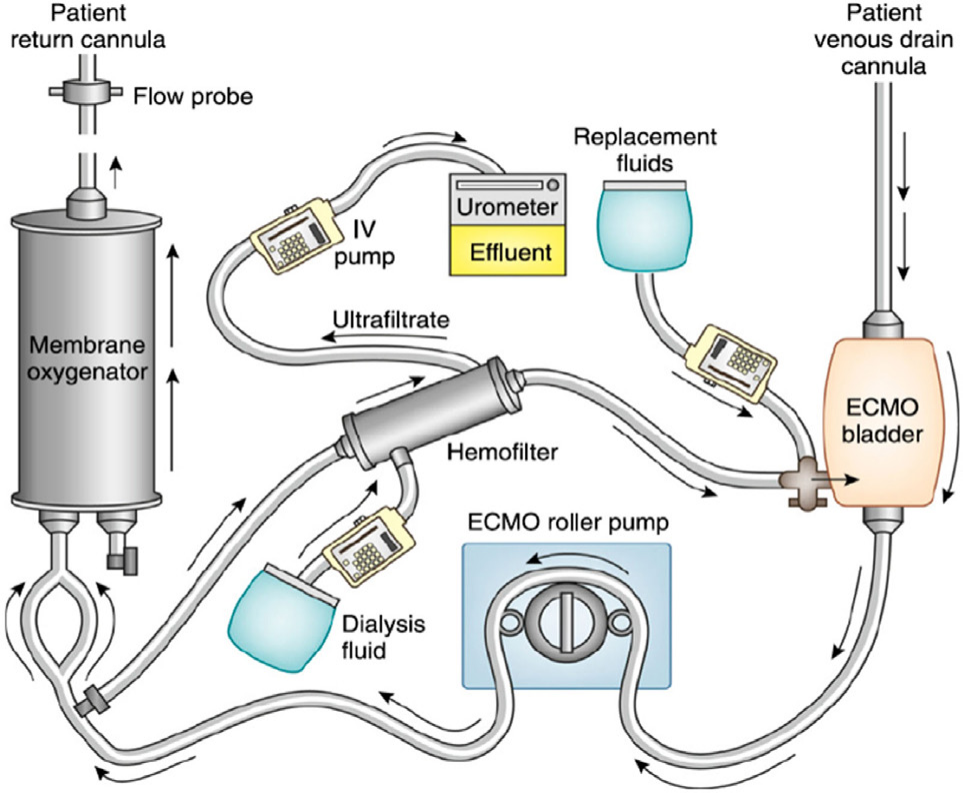
Renal support therapy using an in-line hemofilter with extracorporeal membrane oxygenation (ECMO). Blood from the ECMO circuit is shunted through an in-line hemofilter. The volume of fluid removed by the hemofilter, the ultrafiltrate, can be controlled using an intravenous pump. A filter replacement fluid or dialysis fluid can be used for additional solute clearance. Reprinted with permission from Askenazi et al. (19).

The addition of a hemofilter into the ECMO circuit is relatively simple and cost-effective. The inlet of the filter is connected after the ECMO pump and its outlet is reconnected to the proximal limb of the ECMO circuit. The volume of replacement fluid, dialysis, and effluent fluid is controlled *via* an intravenous infusion pump (Figure 1). One of the drawbacks of this technique is the potential inaccuracy of the amount of volume being delivered and removed with intravenous infusion pumps (31, 32), and more precise methods require substantial increase in bedside workload. Another potential pitfall of this method is the lack of monitoring of the pressures in the hemofiltration circuit, which can lead to a lag time in detection of clotting and/or rupture of the filter (31, 33).

There is evidence that connecting a CRRT device to an ECMO circuit can provide more accurate fluid management. Several techniques have been described for the attachment of a commercially available CRRT device to an ECMO circuit. Santiago et al. described the addition of a CRRT device in series with the ECMO circuit (33). The authors tested their system in a closed circuit, an experimental animal study, and finally in a clinical trial with six children. They found no significant change in the pressures of the ECMO circuit with the introduction of the CRRT device. Other authors have described the addition of a CRRT device in parallel with the ECMO circuit (34), arguing that, in doing so, there is a reduction in blood flow resistance and turbulence after the centrifugal pump. A recent study in a simulated neonatal patient showed no change in the hemodynamic performance using a CRRT circuit in a variety of positions when a centrifugal pump was used. In the case of the roller head pump, the position in which the CRRT device did not have an effect on the hemodynamics of the circuit was in series with the circuit after the pump and before the membrane oxygenator (35). The type of ECMO pump will, therefore, influence the position of the CRRT device. With a centrifugal pump, the device should be connected after the ECMO pump because of the risk of air entrapment due to the negative pressure generated by centrifugal pumps. Regardless of the type of pump, the return blood from the CRRT device should be returned prior to the oxygenator to reduce the risk of air or clot being sent to the patient (19).

While ECMO patients with AKI and/or fluid overload have increased mortality, there has been some concern that the use of CRRT in ECMO patients could increase the risk of developing chronic renal failure. However, these concerns have not been substantiated. Paden et al. showed that in the absence of primary renal disease, 96% of patients had full renal recovery (36). The pathophysiology of AKI in this subset of patients is complex and results from a multitude of factors often working synergistically. Further research into the etiology of AKI and preventive strategies are needed. While most contemporary staging of AKI is based on a change in creatinine from baseline, serum creatinine is an imprecise reflection of renal function, and biomarkers such as NGAL may better reflect renal impairment (37). The use of more accurate biomarkers could better identify patients with AKI and, therefore, the more rapid initiation of CRRT on ECMO. A recent study in which CRRT was implemented in all neonates within 48 h of ECMO cannulation demonstrated a decreased weight gain at the time of CRRT initiation and quicker resolution of fluid overload. However, it did not show significant improvement in duration of ECMO support or mortality (38). A summary of pediatric studies of ECMO and RST is presented in Table 1.

**Table 1:** Pediatric studies of ECMO and RST.

Study	*N*	Study design	Outcome
Selewski et al. (30)	756	Multi-center retrospective cohort study of fluid overload and associated outcomes in neonatal and pediatric patients requiring ECMO	The degree of fluid overload at ECMO initiation and peak fluid overload both predicted hospital mortality. In survivors, the degree of fluid overload at ECMO initiation and peak fluid overload on ECMO predicted the duration of ECMO support. A total of 50.4% of patients received RST

Fleming et al. (12)	832	Multi-center retrospective cohort study of AKI and associated outcomes in neonatal and pediatric patients requiring ECMO	AKI was present in 60–74% of ECMO patients, and it was observed by 48 h of ECMO support in 86–93% of these patients. AKI was associated with a longer duration of ECMO and increased hospital mortality. A total of 47% of patients received RST during ECMO

Selewski et al. (29)	53	Retrospective chart review of neonatal and pediatric patients requiring RST during ECMO	The overall intensive care unit survival was 34% for patients requiring RST during ECMO. Median fluid overload at initiation of RST was significantly lower in survivors versus non-survivors (24.5 vs. 38%, *p* = 0.035)

Askenazi et al. (15)	9,903	Retrospective cohort study of neonatal and pediatric ECMO patients without cardiac disease	The adjusted OR for mortality for neonatal patients with AKI was 3.2 (*p* < 0.0001) and 1.9 for those requiring RST (*p* < 0.0001). The adjusted OR for mortality for pediatric patients with AKI was 1.7 (*p* < 0.001) and 2.5 (*p* < 0.0001) for those requiring RST

Blijdorp et al. (28)	61	Retrospective case-comparison of neonates receiving pre-emptive CVVH during ECMO	Adding CVVH was associated with decreased time on ECMO, decreased time to extubation, decreased blood transfusions, and decreased cost per ECMO run

Hoover et al. (14)	52	Retrospective case-matched study of pediatric patients receiving ECMO with CVVH to those receiving ECMO without CVVH	The use of CVVH with ECMO was associated with improved fluid balance, improved nutrition, and decreased use of diuretics

*ECMO, extracorporeal membrane oxygenation; RST, renal support therapy; AKI, acute kidney injury; OR, odds ratio; CVVH, continuous venovenous hemofiltration*.

## Therapeutic Plasma Exchange (TPE)

Therapeutic plasma exchange is a technique typically carried out *via* a centrifugal device to separate and remove plasma from whole blood or with the use of a semipermeable membrane that separates plasma from whole blood. The removed plasma volume is then replaced, and although the composition is not standardized, typical replacement fluids include varied amounts of normal saline, human albumin, and fresh frozen plasma. As the centrifugal pump separates blood components based on density, TPE is non-selective, but carries the potential benefit of removing pro-inflammatory mediators, antibodies, and cytokines (39, 40). Published by the American Society for Apheresis, guidelines offer current literature as well as category and grade recommendations on conditions amenable to TPE (41). Although prior evidence has shown TPE to be effective for the treatment of thrombotic thrombocytopenic purpura (TTP) (42, 43) and Guillain–Barre syndrome (44), recent studies have sought to evaluate the utility of TPE in the setting of sepsis and thrombocytopenia-associated multiple organ failure (TAMOF) (39, 40, 45–47).

Thrombocytopenia-associated multiple organ failure is a syndrome described in critically ill children, and like TTP, it has been associated with decreased levels of a disintegrin-like and metalloprotease with thrombospondin (ADAMTS-13), which can result in von Willebrand factor-mediated thrombotic microangiopathy and confers an increased risk of mortality (40, 45). Nguyen et al. demonstrated that levels of ADAMTS-13 activity were increased after TPE in children with TAMOF and was associated with improvement in organ dysfunction. Their prospective randomized controlled trial also demonstrated reduced ADAMTS-13 activity level when TPE was stopped in three patients and return of ADAMTS-13 activity when it was reinstituted in two of those patients (40). In 2014, Rimmer et al. published a meta-analysis of four trials of plasma exchange in patients with sepsis that included a total of 194 patients. Of the four trials, one included only adult patients, one included adult and pediatric patients, and two included only pediatric patients. This review showed a significant reduction of all-cause mortality in adults after TPE, but the trend in children was not statistically significant (48). A single-center retrospective study of 14 pediatric ECMO patients with sepsis and TAMOF demonstrated an improvement in Organ Failure Index (OFI) and Vasoactive-Inotropic Scores (VIS) after TPE. The treatment group had an estimated survival rate of 40% (based on OFI) but overall survival rate of 71.4% after treatment with conventional therapy and plasma exchange. They also noted a trend toward improved survival compared to the historical control group, which had a survival rate of 50%. Additionally, through subgroup analyses, a trend toward greater improvement in organ recovery (as determined by change in OFI) and improved hemodynamic status (as determined by VIS) was noted in patients who received TPE earlier in their hospital course, although this was not statistically significant (47).

## Leukopheresis

Severe pertussis is a dreaded disease in young infants, with hypoxia, pulmonary hypertension, and cardiopulmonary collapse that can be refractory to traditional management and confers a high mortality despite aggressive interventions. The pathophysiology of the severe hypoxia and pulmonary hypertension that is seen in these infants is poorly understood. However, based on postmortem studies showing leukocyte thrombi in the pulmonary vasculature and the correlation between degree of hyperleukocytosis and poor outcomes, the hypothesis that viscosity and hyperleukocytosis are major players in this disease process has been brought forward (49, 50). In 2004, Romano et al. conducted the first double volume exchange transfusion as a means for leukodepletion in a 3 months old with severe pertussis on mechanical ventilation with escalating oxygen requirements and evidence of pulmonary hypertension on echocardiogram (51). They saw improvement in oxygenation within a few hours of leukopheresis. Two years later, Grzeszczak et al. first described successful leukopheresis in a 5-week-old patient with severe pertussis necessitating ECMO support and showed dramatic temporal association between leukopheresis initiation and cardiovascular function (49).

Since then, there have been several case reports and case series published, the largest by Rowlands et al. where a comparison was made between patients treated prior to and after the adoption of aggressive leukopheresis (50). The patients requiring ECMO support underwent leukopheresis by adding a white blood cell (WBC) filter in the bridge of the ECMO circuit while priming the circuit, and after ECMO flow was established, opening flow to the bridge (approximately 100 mL/min) until the WBC count approached 15,000/μL. The patients not requiring ECMO support underwent exchange transfusion *via* arterial and central venous lines using 4.5% albumin and packed red blood cells to achieve a WBC count of less than 50,000/μL. The authors found a trend toward higher survival after implementation of aggressive leukopheresis in both the ECMO and non-ECMO supported patients, yet, their results were not statistically significant. However, their quoted 20% mortality for patients requiring ECMO and treated with leukopheresis is significantly lower than the 70% mortality reported in an ELSO database analysis of infants with severe pertussis requiring ECMO support in 2003 (52). More recent reports still describe a high mortality for infants with severe pertussis requiring ECMO support; reports from California in 2015 (53) and Australia and New Zealand in 2016 (54) found a mortality of 98 and 75%, respectively, suggesting that the improved survival seen in Rowlands’s second study period does not reflect improvement in overall care of these infants. The degree of leukocytosis necessitating leukopheresis has not been studied, although the authors from Rowlands et al. recommend leukopheresis for patients on ECMO with WBC counts above 50,000/μL. A recent retrospective, multicenter study of infants requiring ECMO for pertussis showed a survival of 28%. Younger age, lower PaO_2_/FiO_2_ ratio, use of vasoactive infusions, pulmonary hypertension, and a decreased time from intubation to need for ECMO, were all associated with higher mortality. However, leukopheresis was independently associated with increased survival in this patient population [OR 3.36 (1.13–11.68); *p* = 0.03] (55).

## Adsorptive Therapies

Adsorptive therapies have developed as a means of attenuating the effects of severe septic shock. Adsorptive cartridges can be integrated into extracorporeal circuits with the intention of removing endotoxin or inflammatory mediators from systemic circulation (56). This technique allows for the use of polymyxin B, an antibiotic capable of binding and neutralizing endotoxin, but whose systemic use is generally limited due to its toxicity (57, 58). Polymyxin B hemoperfusion (PMX-HP) has been used for over 20 years in Japan with the majority of the literature based on studies of adult patients.

One such study used a national database in Japan to conduct a retrospective analysis of PMX-HP in adults with abdominal septic shock. This review found no significant difference in 28-day mortality between the groups that received PMX-HP in addition to conventional therapies versus conventional therapies alone (58). These findings were consistent with a multicenter randomized control trial in France that also found PMX-HP with conventional therapy compared to conventional therapy alone had no significant difference in the 28-day mortality of patients with septic shock due to peritonitis (56). A systematic review of five trials studying PMX-HP in patients with septic shock noted improvement in mean arterial blood pressures, PaO_2_/FiO_2_ ratios, and decreased mortality, although the authors cautioned that few of these studies were planned or powered to assess mortality (59). While the efficacy of PMX-HP in adults with sepsis is undergoing investigation, there are even fewer studies examining its use in pediatric populations. Thus far, the use of adsorptive therapies in conjunction with ECMO has been limited to case reports (60–62).

## Extracorporeal Liver Support

Extracorporeal liver support has been used as a bridge to recovery or to transplant in patients with acute or acute on chronic liver failure. The types of artificial extracorporeal liver support include single-pass albumin dialysis (SPAD), molecular adsorbent recirculating system (MARS^®^; Gambro, Germany), and the Prometheus^®^ fractionated plasma separation and adsorption system (Fresenius, Germany). SPAD can be performed using a standard continuous renal replacement device. The patient’s blood flows through a standard hemofilter and dialyzate containing albumin flows counter-current to the blood to allow for the removal of protein bound molecules that are not removed with standard renal support therapies, and the used dialysate fluid is then discarded. In a similar fashion, liver support with the MARS device consists of blood flow through a hemofilter with a countercurrent albumin solution that allows for the removal of protein bound toxins. The used albumin solution then undergoes dialysis to remove water soluble toxins and then passes through an anion exchanger resin adsorber and a charcoal absorber. The replenished albumin is then returned to the primary circuit to be utilized again. With the Prometheus system, the albumin is selectively filtered from the patient’s blood, and this albumin enriched plasma is sent through a resin adsorber column and then to an anion exchanger adsorber column to remove toxins that are bound to the albumin. After passing through the two adsorber columns, this cleansed albumin solution returns to the primary circuit to undergo removal of water soluble compounds *via* a hemofilter and conventional dialysis (63, 64).

In a case series of adult ECMO patients with severe hyperbilirubinemia, five patients were supported with MARS in addition to venovenous ECMO. Based on historic controls of ECMO patients with severe hyperbilirubinemia, there would be no expected survivors. However, 40% of the patients supported with MARS survived (65). In a single center, retrospective study of adult ECMO patients with acute liver failure, patients supported with ECMO and MARS therapy had a survival to weaning off ECMO of 64% versus a survival to weaning off ECMO of only 21% in the ECMO and standard medical therapies group (*p* = 0.02). There was a trend in 30-day survival after ECMO in the MARS therapy group versus the standard therapy group (43 versus 14%), but this was not statistically significant (66).

## Conclusion

As ECMO continues to evolve, we will continue to push the limits of the support that can be provided with increasing numbers of higher acuity patients. We continue to support critically ill patients while waiting for organ recovery, or when organ recovery is not possible, to bridge to organ transplantation. There are promising results for the use of other organ support therapies in conjunction with ECMO. However, these results must be interpreted with caution, as there is a lack of prospective, randomized studies looking at the use these therapies with ECMO.

## Author Contributions

BB, MC, and JD made substantial contributions to the writing and revision of this manuscript. Each author approved of the final version of this manuscript.

## Conflict of Interest Statement

The authors declare that the research was conducted in the absence of any commercial or financial relationships that could be construed as a potential conflict of interest.
